# Effects of neuronal adaptation currents on network-based spike rate oscillations

**DOI:** 10.1186/1471-2202-14-S1-P300

**Published:** 2013-07-08

**Authors:** Moritz Augustin, Josef Ladenbauer, Klaus Obermayer

**Affiliations:** 1 Neural Information Processing Group, Berlin Institute of Technology, Berlin, Germany; 2 Bernstein Center for Computational Neuroscience Berlin, Berlin, Germany

## 

Local field potential in-vivo recordings often show oscillations with frequencies ranging from < 1 Hz to 100 Hz. The underlying mechanism could change across frequency bands but likely involves network interactions such as recurrent synaptic excitation-inhibition loops. Particularly fast rhythmic activity in the beta and gamma range is thought to be caused by synaptic inhibition [1]. Although these oscillations primarily depend on synaptic properties, their frequency is significantly influenced by the passive membrane characteristics of single cells [2]. In addition, neuronal membranes typically contain slowly deactivating voltage-dependent as well as calcium-activated potassium channels both which mediate spike rate adaptation.

Here we investigate how the dynamics of such neuronal adaptation currents contribute to synaptically generated spike rate oscillations and resonance properties in recurrent networks of excitatory and inhibitory neurons. Based on a network of sparsely delay-coupled adapting spiking model neurons we take a mean-field approach using the Fokker-Planck equation to analyze oscillatory network activity. In the limit of slow adaptation timescales we obtain population spike rates and membrane potential distributions [3]. To allow for a linear stability analysis we derive a low-dimensional system of ordinary differential equations from the Fokker-Planck mean-field model. This system effectively describes the activity of the network while retaining the features of the spiking neurons (i.e. the model parameters).

**Figure 1:**
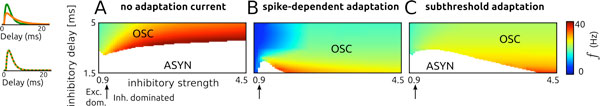
**Influence of synaptic inhibition and neuronal adaptation on network-based oscillations**. A-C: Existence of oscillatory network states (OSC) and corresponding frequencies f as a function of the strength and timescale of synaptic inhibition for networks without adaptation (A), with increased spike-dependent adaptation (B) and increased sub-threshold adaptation (C). Asynchronous network states (ASYN) are indicated by white regions. Arrows mark balance of recurrent excitation and inhibition. On the left the delay distributions (green: excitation, orange: inhibition) corresponding to faster (bottom) and slower inhibition (top) are shown.

For constant external input we find that fast oscillations stabilize if recurrent inhibition is slower than excitation. The oscillation frequency increases with increasing inhibitory synaptic efficacy and decreasing inhibitory delay. Neuronal adaptation facilitates such network-based oscillations for fast synaptic inhibition and leads to decreased frequencies (see Figure 1). Additionally we characterize chaotic spike rate dynamics, which is produced by the interplay of spike-dependent adaptation and slow synaptic inhibition. For oscillatory external input, adaptation currents lead to amplification of a narrow band of frequencies and cause phase advances for low frequencies in addition to phase delays at higher frequencies. Our results highlight the role of intrinsic neuronal adaptation properties in determining the collective rhythms originating from network interactions.
